# The clinical efficacy and safety of dapagliflozin in patients with diabetic nephropathy

**DOI:** 10.1186/s13098-022-00815-y

**Published:** 2022-03-29

**Authors:** Ying Huang, Wen Lu, Hongyun Lu

**Affiliations:** Department of Endocrinology, Zhuhai People’s Hospital, Zhuhai Hospital Affiliated With Jinan University, No.79 Kangning Road Zhuhai, Guangdong, 519000 China

**Keywords:** Diabetic nephropathy, Dapagliflozin, Sodium-glucose cotransport protein 2 inhibitor, Renal function

## Abstract

**Objective:**

To investigate the clinical efficacy and safety of dapagliflozin in the treatment of diabetic nephropathy (DN).

**Methods:**

A total of 120 DN patients admitted to our hospital from June 2017 to March 2020 were divided into control and experimental groups, with 60 cases in each group. The control group received valsartan, and the experimental group received dapagliflozin for 3 months. Body mass index (BMI), hemoglobin A1c (HbA1c), serum creatinine (sCr), uric acid (UA), urine microalbumin (uMA), urine creatinine (uCr), and bilateral kidney function were compared before and after treatment, and adverse reactions in both groups were observed. Serum interleukin-6 (IL-6) and tumor necrosis factor-α (TNF-α) levels were also evaluated.

**Results:**

After treatment, except for BMI in the control group, all indexes in both groups were significantly improved. The BMI, HbA1c, sCr, UA, and uMA/uCr ratios of the experimental group were lower than those of the control group. Serum albumin (sAlb) levels were increased in both groups, and the experimental group showed a significant difference compared with the control group. Estimated glomerular filtration rate (eGFR) levels were increased in both groups, and the experimental group was higher than the control group, with no significant differences. Serum IL-6 and TNF-α levels in both groups were lower, and the experimental group was significantly lower than the control group. No serious adverse reactions were observed in either group.

**Conclusion:**

The efficacy of dapagliflozin was demonstrated by its ability to improve diabetes, prevent nephropathy exacerbation, and reduce symptomatic reactions. The low rate of adverse reactions makes dapagliflozin a very safe medication.

## Introduction

Diabetic nephropathy (DN) is one of the common microvascular complications of diabetes and is characterized by albuminuria and glomerular filtration rate (GFR) reduction [1]. Hyperglycemia and glucose metabolites such as advanced glycation end products (AGEs) have long been regarded as initial factors of DN, which promote the loss of podocytes, the hyperfiltration of endothelial cells, the expansion of mesangial cells, and the thickening of the glomerular basement membrane. [2]. Although many therapies have been used to improve DN, the quality of life of an increasing number of patients is severely impacted [3]. As a result, more novel therapeutic strategies are needed for DN treatment.

Recent advances emphasized the therapeutic value of sodium-glucose cotransporter-2 inhibitors (SGLT2i), which reduced the risk of progression of renal disease by 45% [4]. Dapagliflozin is an SGLT2i that has been shown to decrease glycated hemoglobin (HbA1c) by promoting urinary glucose excretion, body weight, systolic blood pressure, and albuminuria [5]. A large cardiovascular outcome trial demonstrated that dapagliflozin also significantly reduced the risks of heart failure and progression of chronic kidney disease in people with type 2 diabetes [6]. Some recent studies have focused on the DN therapeutic effect of dapagliflozin. It was reported that ticagrelor and dapagliflozin have additive effects on ameliorating diabetic nephropathy in mice with type-2 diabetes mellitus [7]. Additionally, dapagliflozin treatment improved histopathological examinations, inflammatory and apoptotic markers in a dose-dependent manner when compared to diabetic vehicles, implying that dapagliflozin may have renoprotective effects, which are promising in diabetic patients with nephropathy. [8]. Furthermore, dapagliflozin attenuates early markers of diabetic nephropathy in fructose-streptozotocin-induced diabetes in rats [9]. However, most research on the DN therapeutic effect of dapagliflozin has been performed in mice or rat models, and the clinical data are minimal.

Thus, in this study, we conducted a clinical study on the efficacy and safety of dapagliflozin in patients with diabetic nephropathy by evaluating body mass index (BMI), hemoglobin A1c (HbA1c), serum creatinine (sCr), uric acid (UA), urine microalbumin (uMA), urinary creatinine (uCr), bilateral kidney function, and the occurrence of adverse reactions.

## Materials and methods

### General information

A total of 120 Chinese patients with DN in our hospital (Zhuhai People’s Hospital) from June 2017 to March 2020 were selected and randomly divided into the control group (n = 60) and experimental group (n = 60). Power analysis was used to calculate sample size, and patients were randomly assigned to one of two groups in a 1:1 ratio. The control group was treated with valsartan, and the experimental group was treated with dapagliflozin. The primary characteristics, including age, sex, course of disease (years), BMI (kg/m^2^), HbA1c (%), sCr (μmol/L), UA (μmol/L), and urinary amylase/urinary creatinine ratio uMA/uCr (μg/mg), were compared before and after treatment.

### Inclusion and exclusion criteria

Inclusion criteria: (i) age between 18 and 70 years; (ii). patients who met the diagnostic criteria for diabetes mellitus published by the World Health Organization (WHO) in 2021, 1999 and had stable glucose control (less than 10% change in the previous 2 months); (iii) persistent albuminuria (≥ 30 mg/24 h) or eGFR < 60 ml/min/1.73 m^2^; glycated HbA1c was 7.0% ~ 11.0%, and the patients with inadequate blood glucose control but good compliance were treated with oral hypoglycemic agents or subcutaneous insulin injections to control blood glucose; (iv) individuals with normal cognitive function and written informed consent.

Exclusion criteria: (i) patients with non-diabetic nephropathy, such as glomerulonephritis and urinary tract infection; (ii) patients with severe cardiovascular and cerebrovascular diseases; and heart failure (New York Heart Association functional class III-IV); (iii) liver dysfunction (aspartate transaminase and alanine transaminase levels > 100 IU/L); (iv) patients with acute complications of diabetes, such as diabetic ketoacidosis and diabetic hyperosmolar coma; patients with malignant tumors; patients with a previous history of SGLT-2i medication and patients allergic to angiotensin receptor blocker (ARB) drugs.

### Methods

Routine nursing followed the standard management of diabetes, including daily diet, physical exercise, and blood glucose monitoring. Based on oral hypoglycemic drugs or subcutaneous insulin injections to control blood glucose, the control group was given 80 mg valsartan (Beijing Novartis Pharmaceutical Co., Ltd., approval number: Guoyao Zhun H20040217, specification: 80 mg/tablet) twice a day, and the experimental group was given 10 mg of dapagliflozin (AstraZeneca Pharmaceutical Co., Ltd., batch number: H20170040, specification: 10 mg/tablet) once a day. Both groups received treatment for 3 months.

### Diabetic nephropathy- and symptomatic reaction-associated indexes

The sAlb was detected by the bromocresol green (BCG) method; the estimated glomerular filtration rate (eGFR) was affected by the level of sAlb, and an automatic biochemical analyzer detected blood glucose and renal function. The eGFR was calculated using the Modification of Diet in Renal Disease (MDRD) formula.

The BMI of the two groups before and after treatment was calculated. HbA1c, sCr, UA, uMA, and uCr were detected by the Abbott chemiluminescence method, and the uMA/uCr ratio was calculated.

Adverse reactions (such as hypoglycemia, hypotension, dizziness, and urinary tract infection) were recorded, and the total incidence of adverse reactions was calculated.

Inflammatory indexes, including interleukin-6 (IL-6) and tumor necrosis factor-α (TNF-α), were detected before and after treatment. Radioimmunoassays were used to detect IL-6 and TNF-α.

### Statistical analysis

All data were analyzed by SPSS 22.0. The measurement data are expressed as the mean ± standard deviation (x ± s) and were compared with a t-test. The count data were expressed as a percentage (%) and compared with the χ^2^ test. *P* < 0.05 was considered statistically significant.

## Results

### Comparison of the primary characteristics

The average age of the control group was 55.67 ± 11.46 years, 36 males and 24 females, and the average course of the disease was 9.65 ± 2.55 years; the average age of the experimental group was 56.21 ± 11.18 years, 34 males and 26 females, and the average course of the disease was 10.04 ± 2.31 years. There was no significant difference in age, sex, course of the disease, or other general information between the two groups (*P* > 0.05), which was comparable, as shown in Table 1.

**Table 1:** Comparison of primary clinical data between the two groups before treatment

Groups	Control group	Experimental group	t/χ^2^	*P*
Age (years)	55.67 ± 11.46	56.21 ± 11.18	2.108	> 0.05
Gender (male/female)	36/24	34/26	2.725	> 0.05
Course of disease (years)	9.65 ± 2.55	10.04 ± 2.31	2.611	> 0.05
BMI (kg/m^2^)	25.94 ± 2.51	26.13 ± 2.35	2.002	> 0.05
HbA1c (%)	9.36 ± 1.44	9.31 ± 1.72	2.514	> 0.05
sCr (μmol/L)	140.21 ± 34.84	143.08 ± 36.33	2.753	> 0.05
UA (μmol/L)	366.10 ± 68.54	371.40 ± 69.59	2.647	> 0.05
uMA/uCr (μg/mg)	251.52 ± 57.29	256.55 ± 58.10	2.435	> 0.05
Medications				
Insulin	54	57	0.04213	> 0.05
Metformin	56	58	0.018	> 0.05
Sulphonylureas	6	4	0.3693	> 0.05

*BMI* body mass index, *HbA1c* hemoglobin A1c, *sCr* serum creatinine, *UA* uric acid, *uMA/uCr* microalbumin to creatinine ratio

After treatment, in addition to the BMI of the control group, all the other indexes of both groups were significantly improved (*P* < 0.05). After the treatment, the BMI, HbA1c, sCr, UA, and uMA/uCr ratio of the experimental group were lower than those of the control group (*P* < 0.05), as shown in Table 2. These results indicate that dapagliflozin improves the basic indicators of DN patients.

**Table 2:** Comparison of the basic indicators between the two groups after treatment

Groups	N		BMI (x ± s, kg/m^2^)	HbA1c (x ± s, %)	sCr (x ± s, μmol/L)	UA (x ± s, μmol/L)	uMA/uCr (μg/mg)
Control group	60	Before treatment	25.94 ± 2.51	9.36 ± 1.44	140.21 ± 34.84	366.10 ± 68.54	251.52 ± 57.29
		After treatment	25.3 ± 2.21	7.37 ± 0.91	121.63 ± 25.40	345.54 ± 61.22	196.57 ± 45.36
Experimental group	60	Before treatment	26.13 ± 2.35	9.31 ± 1.72^*^	143.08 ± 36.33^*^	371.40 ± 69.59^*^	256.55 ± 58.10^*^
		After treatment	24.08 ± 2.33^*#^	6.73 ± 0.56^*#^	104.10 ± 24.68^*#^	321.57 ± 60.91^*#^	173.47 ± 46.16^*#^

*BMI* body mass index, *HbA1c* hemoglobin A1c, *sCr* serum creatinine, *UA* uric acid, *uMA/uCr* microalbumin to creatinine ratio

^*^indicates *P* < 0.05 compared with before treatment

^#^indicates *P* < 0.05 compared with the control group

### Comparison of sAlb and eGFR levels

Before treatment, there was no significant difference in sAlb or eGFR between the two groups (*P* > 0.05). After treatment, the sAlb levels of the two groups were increased, and experimental group results were significantly higher than those of the control group (*P* < 0.05). The eGFR levels of the two groups were also increased after treatment; however, the experimental group exhibited higher levels than the control group, but the difference was not statistically significant (*P* > 0.05) (Table 3). These results indicate that the levels of sAlb were increased by dapagliflozin treatment.

**Table 3:** Comparison of sAlb and eGFR levels between the two groups

Groups	N	sAlb (g/L)		eGFR[mL/min/1.73m^2^]	
		Before treatment	After treatment	Before treatment	After treatment
Control group	60	31.63 ± 2.24	36.24 ± 3.17	110.08 ± 27.64	111.79 ± 24.72
Experimental group	60	31.15 ± 2.09	39.79 ± 2.58^*#^	111.17 ± 29.22	113.01 ± 26.66

*sAlb* Serum albumin, *eGFR* Estimated Glomerular Filtration Rate

^*^P < 0.05 compared with before treatment

^#^*P* < 0.05 compared with the control group

### Comparison of serum inflammatory factors before and after treatment

Before treatment, there was no significant difference in serum IL-6 and TNF-α levels between the two groups (*P* > 0.05). On the 1st day after the operation, the levels of serum IL-6 and TNF-α in both groups were lower than those before treatment, and the levels in the experimental group were significantly lower than those in the control group (*P* < 0.05) (Table 4). These results indicate that the serum inflammatory factors were decreased after dapagliflozin treatment.

**Table 4:** Comparison of serum inflammatory factors between the two groups before and after treatment

Groups	N	IL-6 (pg/ml)		TNF-α (pg/ml)	
		Before treatment	After treatment	Before treatment	After treatment
Control group	60	75.77 ± 18.24	49.17 ± 13.68^*^	73.86 ± 26.44	41.57 ± 9.36^*^
Experimental group	60	73.18 ± 17.02	37.43 ± 12.16^*#^	70.84 ± 24.25	32.01 ± 8.21^*#^

^*^P < 0.05 compared with before treatment

^#^*P* < 0.05 compared with the control group

### Comparison of adverse reactions

The patients in the control group were well tolerated, including 2 cases of hypoglycemia, 2 cases of hypotension, and 3 cases of urinary tract infection. There were 3 cases of dizziness and 3 cases of hypotension in the experimental group. There was no significant difference in the total incidence of adverse reactions between the two groups (*P* > 0.05). No other severe adverse reactions occurred in either group (Table 5). These data indicate that dapagliflozin has a low total incidence of adverse reactions.

**Table 5:** Comparison of adverse reactions between the two groups

Groups	N	Hypoglycemia	Hypotension	Dizziness	Urinary tract infection	Incidence of adverse reactions
Control group	60	2	2	0	3	13.33
Experimental group	60	0	3	3	0	10.00

## Discussion

The incidence rate of diabetes and its complications has become increasingly higher because of changes in diet structure. DN is the leading cause of chronic kidney disease (CKD) and renal failure [10]. At present, there is no definite conclusion on the pathogenesis of DN, which is generally believed to be the result of the action of multiple factors, which may be related to abnormal glomerular hemodynamics, increased glomerular pressure, and activation of inflammatory factors. Edema and proteinuria are common clinical symptoms. When they develop to a certain extent, they will cause renal vascular damage due to high blood glucose and blood pressure. A load of renal filtered blood will aggravate and lead to renal disease. Later, they are likely to develop into uremia [11]. Therefore, the early treatment of the disease is very important, and the main principle of treatment is to control blood sugar and reduce the burden on the kidney [12].

Sodium-glucose cotransporter (SGLT) is expressed in the renal proximal convoluted tubules and can be divided into SGLT-1 and SGLT-2i subtypes. The SGLT-2i receptor is a glucose transporter with high capacity, high transport capacity, and low affinity. It is expressed in the S1 and S2 segments of proximal convoluted tubules and mainly undertakes glucose reabsorption [13, 14]. In diabetic patients, the upregulation of SGLT-2i helps to maintain hyperglycemia, while inhibition of SGLT-2i can effectively improve blood glucose control. SGLT-2i can reduce blood glucose by inhibiting the reabsorption of sodium and glucose by renal proximal convoluted tubules, increasing the excretion of urine glucose, increasing the function of islet β cells, improving insulin resistance, and greatly reducing renal metabolic disorders caused by chronic hyperglycemia [15, 16]. In addition, SGLT-2i also increases the excretion of sodium in renal tubules, resulting in a diuretic effect, that reduces the volume of body fluid and blood pressure, thus reducing glomerular ultrafiltration [17]. Dapagliflozin is a representative SGLT-2i drug that has been widely used in patients with diabetes [18]. The results of this study showed that after 3 months of treatment with dapagliflozin, the BMI, HbA1c, sCr, UA, and uMA/uCr ratio of the patients decreased, indicating that dapagliflozin can effectively control glucose and protect the kidney, which is consistent with the results of previous studies [19]. After treatment, the indexes of the experimental group were lower than those of the control group, indicating that dapagliflozin is more effective than valsartan in the treatment of DN. In addition, the sAlb and eGFR levels of both groups increased after treatment, and the sAlb levels in the study group were significantly higher than those of the control group. Although there was no significant difference in eGFR levels between the two groups. The possible reason is that in the early stage of diabetic nephropathy, sodium-glucose cotransporters (SGLTs) can increase the reabsorption of sodium ions and fluid in renal proximal convoluted tubules. This reabsorption can lead to glomerular hyperfiltration by reducing the concentrations of Na and K in dense plaques and increasing the GFR is caused by tubule feedback. As an inhibitor of SGLT-2, dapagliflozin can improve the glomerular hyperfiltration state and protect the kidney [20–22].

The traditional pathogenesis of DN is the dual effects of hemodynamic changes and severe persistent hyperglycemia. Recent studies have shown that DN is a chronic low-grade inflammatory state, and inflammation-related molecules and pathways play an important role in the process of early DN, indicating that targeted inflammation may be one of the methods to treat DN [23]. Krishan et al. [24] showed that high mobility group box-1 (HMGB1) was closely related to the occurrence and development of DN and positively correlated with TNF-α and IL-6. In addition, dapagliflozin may have an additional anti-inflammatory effect on the kidneys, which is independent of blood glucose and blood pressure [25]. Urinary excretion of renal tubular injury markers and inflammatory markers (such as IL-6) decreased in type 2 diabetes (T2DM) patients after treatment with dapagliflozin [26]. This study showed that the inflammatory indexes (TNF-α, IL-6) of the dapagliflozin group were significantly lower than those at baseline after 3 months of treatment, which was consistent with the above results. After 3 months of treatment, although the indexes of both groups improved, the changes in the dapagliflozin group were more significant than those in the valsartan group. Therefore, dapagliflozin has a renal protection function independent of reducing blood glucose, blood pressure, and body weight, which can improve the glomerular and renal tubular function of patients with early DN, reduce the release of TNF-α and IL-6 inflammatory factors, and improve renal function through anti-chronic inflammatory effects.

## Conclusion

In the clinical treatment of patients with diabetic nephropathy, dapagliflozin has a significant effect, which can effectively improve the condition of patients with diabetes, control the aggravation of patients with nephropathy, reduce the symptoms of patients, and have an outstanding curative effect. In addition, the probability of adverse reactions in patients after the medication is low, safe, and effective.

## Data Availability

The datasets generated during and/or analyzed during the current study are available from the corresponding author on reasonable request.

## Abbreviations

AGE: Advanced glycation end
ARB: Angiotensin receptor blocker
BCG: Bromocresol green
BMI: Body mass index
CKD: Chronic kidney disease
DN: Diabetic nephropathy
GFR: Glomerular filtration rate
eGFR: Estimated glomerular filtration rate
IL-6: Interleukin-6
TNF-α: Tumor necrosis factor-alpha
SGLT: Sodium-glucose cotransporter
T2DM: Type 2 diabetes
SGLT: Sodium-glucose cotransporter
MDRD: Modification of Diet in Renal Disease
UA: Uric acid
WHO: World Health Organization
HbA1c: hemoglobin A1c
sCr: Serum creatinine
uMA/uCr: Microalbumin to creatinine ratio
